# Supplementary material for: Anomalous quantized plateaus in two-dimensional electron gas with gate confinement
Source: Nat Commun. 2023 Mar 30;14:1758. doi: 10.1038/s41467-023-37495-9 (PMC10064851; doi:10.1038/s41467-023-37495-9)
Supplement: Supplementary file 1 — supplementary information [file 41467_2023_37495_MOESM1_ESM.pdf]

Supplementary information of

"Anomalous quantized plateaus in two-dimensional electron gas with gate confinement"

Jiaojie Yan[1], Yijia Wu[1], Shuai Yuan[1], Xiao Liu[1], L. N. Pfeiffer[2], K. W. West[2], Yang Liu[1], Hailong Fu[3], X. C. Xie[1,4] and Xi Lin[1,4,5,*]

[1] International Center for Quantum Materials, Peking University, Beijing 100871, China
[2] Department of Electrical Engineering, Princeton University, Princeton, New Jersey 08544, USA
[3] School of Physics, Zhejiang University, Hangzhou 310027, China
[4] CAS Center for Excellence in Topological Quantum Computation, University of Chinese Academy of Sciences, Beijing 100190, China
[5] Interdisciplinary Institute of Light-Element Quantum Materials and Research Center for Light-Element Advanced Materials, Peking University, Beijing 100871, China

* xilin@pku.edu.cn

Supplementary Note 1. Calculations in detail

1.1 $\nu_{\mathrm{II}} > \nu_{\mathrm{I}}$

We assume that regions I and II are both in FQH or IQH state. Their filling factors can be written as $\nu_{\mathrm{I}} = i + \nu_{\mathrm{I}}'$ and $\nu_{\mathrm{II}} = i + \nu_{\mathrm{II}}'$, with i as an integer and $0 < \nu_{\mathrm{I}}' < \nu_{\mathrm{II}}' \leq 1$. If transmitted edge currents obtain equilibration with reflected edge currents in region II, we can write down the equations of each "contact" in Supplementary Fig. 1a according to the Landauer-Buttiker formula[1-3]. Here, contacts labeled "U" and "L" are "virtual contacts", which indicate that edge modes inflow and outflow at these points are in equilibrium and share the same chemical potential.

$$S: \quad I = \frac{e^2}{h}[\nu_{\mathrm{I}} \cdot V_S - \nu_{\mathrm{I}} \cdot V_2]$$

$$1: \quad 0 = \frac{e^2}{h}[\nu_{\mathrm{I}} \cdot V_1 - \nu_{\mathrm{I}} \cdot V_S]$$

$$2: \quad 0 = \frac{e^2}{h}[\nu_{\mathrm{I}} \cdot V_2 - i \cdot V_4 - \nu_{\mathrm{I}}' \cdot V_L]$$

$$3: \quad 0 = \frac{e^2}{h}[\nu_{\mathrm{I}} \cdot V_3 - i \cdot V_1 - \nu_{\mathrm{I}}' \cdot V_U]$$

$$4: \quad 0 = \frac{e^2}{h}[\nu_{\mathrm{I}} \cdot V_4 - \nu_{\mathrm{I}} \cdot V_D]$$

$$D: \quad -I = \frac{e^2}{h}[\nu_{\mathrm{I}} \cdot V_D - \nu_{\mathrm{I}} \cdot V_3]$$

$$U: \quad 0 = \frac{e^2}{h}[\nu_{\mathrm{II}}' \cdot V_U - \nu_{\mathrm{I}}' \cdot V_1 - (\nu_{\mathrm{II}} - \nu_{\mathrm{I}}) \cdot V_L]$$

$$L: \quad 0 = \frac{e^2}{h}[\nu_{\mathrm{II}}' \cdot V_L - \nu_{\mathrm{I}}' \cdot V_4 - (\nu_{\mathrm{II}} - \nu_{\mathrm{I}}) \cdot V_U]$$

And then R_D can be derived as:

$$R_D = \frac{V_1 - V_4}{I} = \frac{h}{e^2} \Big/ \left(i + \frac{\nu_{\mathrm{II}}' \cdot \nu_{\mathrm{I}}'}{2\nu_{\mathrm{II}}' - \nu_{\mathrm{I}}'} \right)$$

which is the scenario we discuss in this work.

1.2 $\nu_{\mathrm{II}} < \nu_{\mathrm{I}}$

If $\nu_{\mathrm{II}} < \nu_{\mathrm{I}}$, edge currents will be partially reflected as they propagate from region I to region II, as shown in Supplementary Fig. 1b. In this case, equations of contacts are shown as follows:

$$S: \quad I = \frac{e^2}{h}[\nu_{\mathrm{I}} \cdot V_S - \nu_{\mathrm{I}} \cdot V_2]$$

$$1: \quad 0 = \frac{e^2}{h}[\nu_{\mathrm{I}} \cdot V_1 - \nu_{\mathrm{I}} \cdot V_S]$$

$$2: \quad 0 = \frac{e^2}{h}[\nu_{\mathrm{I}} \cdot V_2 - \nu_{\mathrm{II}} \cdot V_4 - (\nu_{\mathrm{I}} - \nu_{\mathrm{II}}) \cdot V_1]$$

$$3: \quad 0 = \frac{e^2}{h}[\nu_{\mathrm{I}} \cdot V_3 - \nu_{\mathrm{II}} \cdot V_1 - (\nu_{\mathrm{I}} - \nu_{\mathrm{II}}) \cdot V_4]$$

$$4: \quad 0 = \frac{e^2}{h}[\nu_{\mathrm{I}} \cdot V_4 - \nu_{\mathrm{I}} \cdot V_D]$$

$$D: \quad -I = \frac{e^2}{h}[\nu_{\mathrm{I}} \cdot V_D - \nu_{\mathrm{I}} \cdot V_3]$$

R_D can be derived as:

$$R_D = \frac{V_1 - V_4}{I} = \frac{h}{e^2} \cdot \frac{1}{\nu_{\mathrm{II}}}$$

This is the common situation when measuring devices with lateral confinement, and it looks as if R_D is measuring the Hall resistance in region II. However, this is correct only with the precondition $\nu_{\mathrm{II}} < \nu_{\mathrm{I}}$.

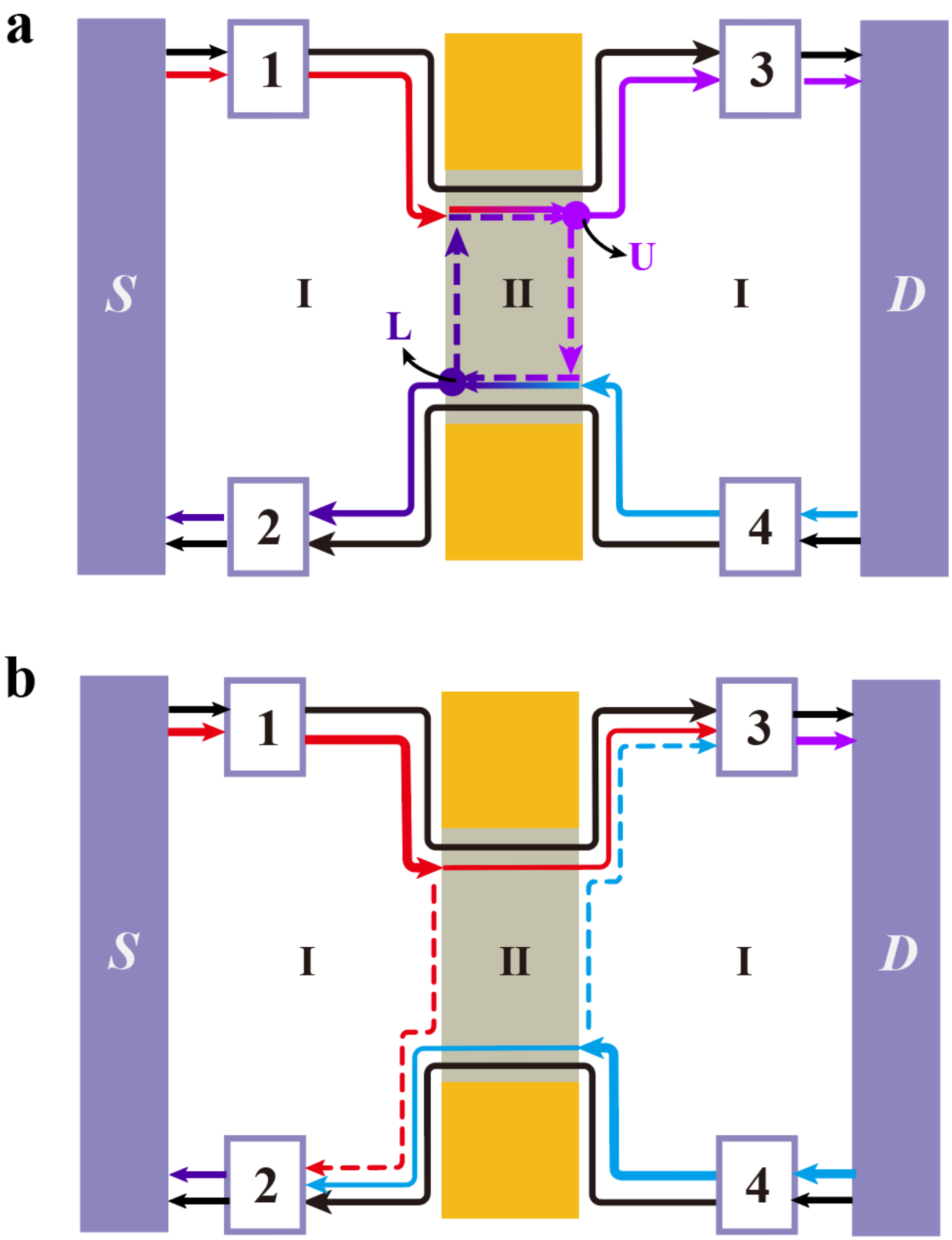

Supplementary Figure 1| Sketch of edge modes propagation and reflection when the filling factor in region II is larger (a) and smaller (b) than region I. a, When $\nu_{\mathrm{II}} > \nu_{\mathrm{I}}$, edge currents propagate in the same direction as that in Fig. 3a in the main text. Edge currents are reflected when propagating from region II towards region I. In region II, mixed edge currents get equilibrium before they reach the interface of different regions. Imaginary contacts labeled as U and L indicate that at these positions, edge modes are equilibrated, and the currents inflow and outflow share the same chemical potential. **b,** When $\nu_{\mathrm{II}} < \nu_{\mathrm{I}}$, edge currents are reflected when propagating from region I to region II. The reflected currents propagate along the interface on the side of region I.

Supplementary Note 2. Typical traces of R_{XY}, R_D, R_{XX}, and R_L

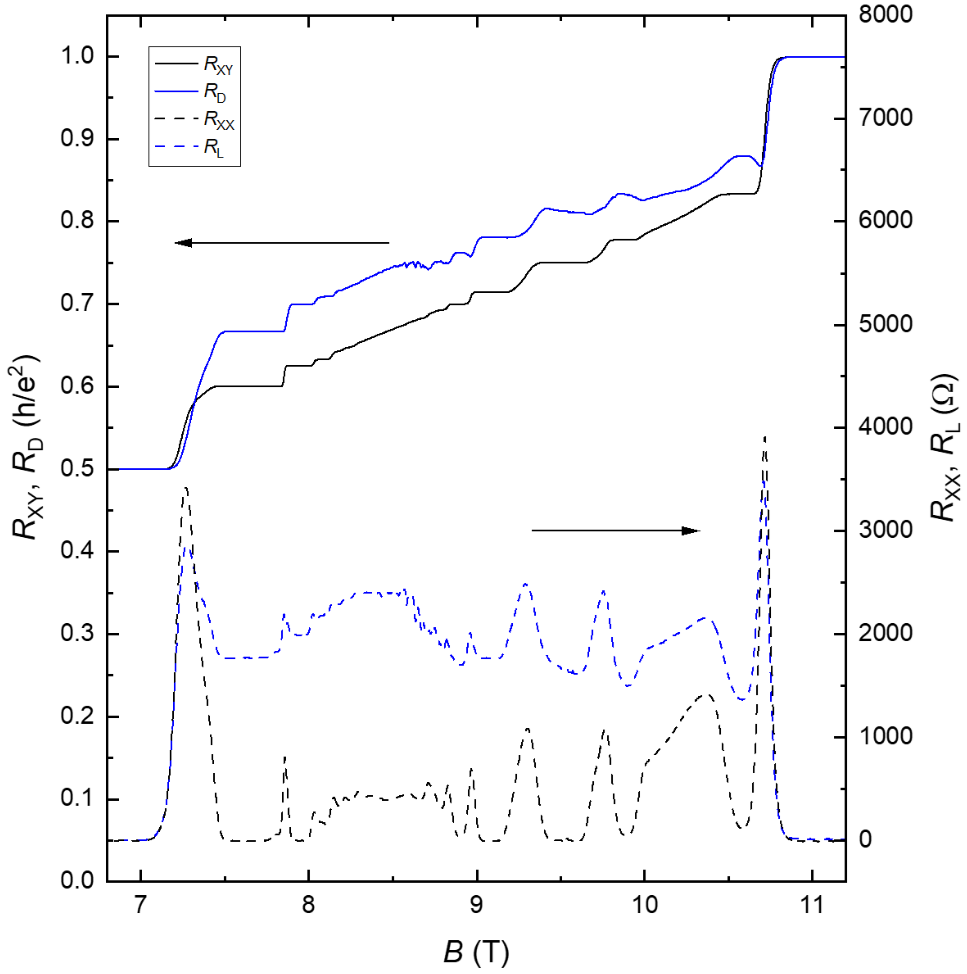

Supplementary Figure 2| Typical traces of R_{XY}, R_D, R_{XX}, and R_L versus magnetic field. R_L is the longitudinal resistance across the confined region and can be measured from contact 1 and 3 or from contact 2 and 4 (Supplementary Fig. 1a). When R_D appears as anomalous plateaus, R_L appears as plateaus with finite values, rather than being zero. Source data are provided as a Source Data file.

Supplementary Note 3. Coexistence of plateaus and their relationship with electron density variation

In the main text, we attribute the appearance of anomalous plateaus to a gate-induced density increase in the confined region. In this section, the relationship between anomalous plateaus and the electron density variation in region II is discussed.

Supplementary Fig. 3a shows R_{D} traces at three different gate conditions in $1 < \nu < 2$. Plateaus can appear together at the same gate voltage, such as the coexistence of $R_{\mathrm{K}}/(3/2)$, $R_{\mathrm{K}}/(10/7)$, $R_{\mathrm{K}}/(9/7)$ and $R_{\mathrm{K}}/(16/13)$ plateaus in the blue trace. This suggests that the emergence of these plateaus share the same origin, which can be explained by an electron density modulation in region II. To make it clear, the relationship between plateaus and $n_{\mathrm{II}}/n_{\mathrm{I}}$ is illustrated in Supplementary Fig. 3b.

The y axis $n_{\mathrm{II}}/n_{\mathrm{I}}$ represents the relative density between regions I and II. $n_{\mathrm{II}}/n_{\mathrm{I}}$ should be larger than 1 in our experiments. The x axis is magnetic field, it corresponds to the filling factor of the open region IQH/FQH state. From the R_{XY} trace, the filling factor range for each IQH/FQH state can be obtained (defined by the plateau from R_{XY}). As a simple estimation, we assume that the filling factor range for each state does not change when density varies. Then we know the filling factor range when region II will enter each IQH/FQH state, and we know ν_{I} and ν_{II} ranges when R_{D} will become plateaus. The filling factor range difference between the regions I and II determines the value of $n_{\mathrm{II}}/n_{\mathrm{I}}$ at different magnetic fields. As consequence, the shapes in Supplementary Fig. 3b are drawn in relationship of $n_{\mathrm{II}}/n_{\mathrm{I}}$ and *B*.

As Hall resistance of region II cannot be measured directly in our devices, we measure R_{D} instead. Anomalous plateaus can coexist at specific $n_{\mathrm{II}}/n_{\mathrm{I}}$ values, as shown by the horizontal dashed lines in Supplementary Fig. 3b. And the three dashed lines correspond to the three R_{D} traces in identical colors in Supplementary Fig. 3a qualitatively.

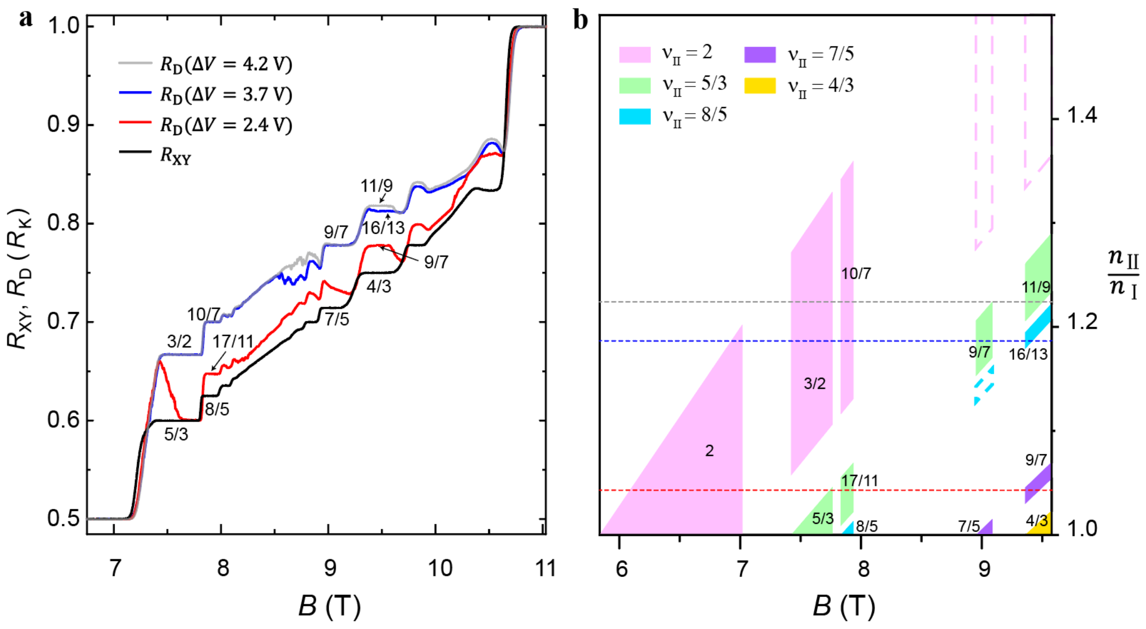

Supplementary Figure 3| Coexistence of plateaus and their relationship with electron density in region II. a, R_D traces in $1 < \nu < 2$ at different gate voltages. Multiple anomalous plateaus can emerge together in one trace. The annealing voltages are -5.5 V (gray) and -5.0 V (blue and red). Source data are provided as a Source Data file. **b,** Phase diagram of anomalous plateaus with n_{II}/n_I and magnetic fields. The shapes suggest conditions when R_D are expected to appear as plateaus, and their colors represent corresponding IQH/FQH states in region II. The expected R_D quantization values are noted in the figure. Filled shapes indicate plateaus observed in our experiments while shapes with dashed boundary lines are plateaus unobserved. The three horizontal dashed lines represent three different densities in region II, and they correspond to three gate voltage conditions in **a**. The positions of the dashed lines are determined qualitatively according to the values of R_D plateaus in **a**.

Supplementary Note 4. Determination of the corresponding fractions for anomalous plateaus

We determine the fraction of each anomalous plateau in Fig. 2 in the main text by comparing the measured decimal to the adjacent fractions with denominators within 100.

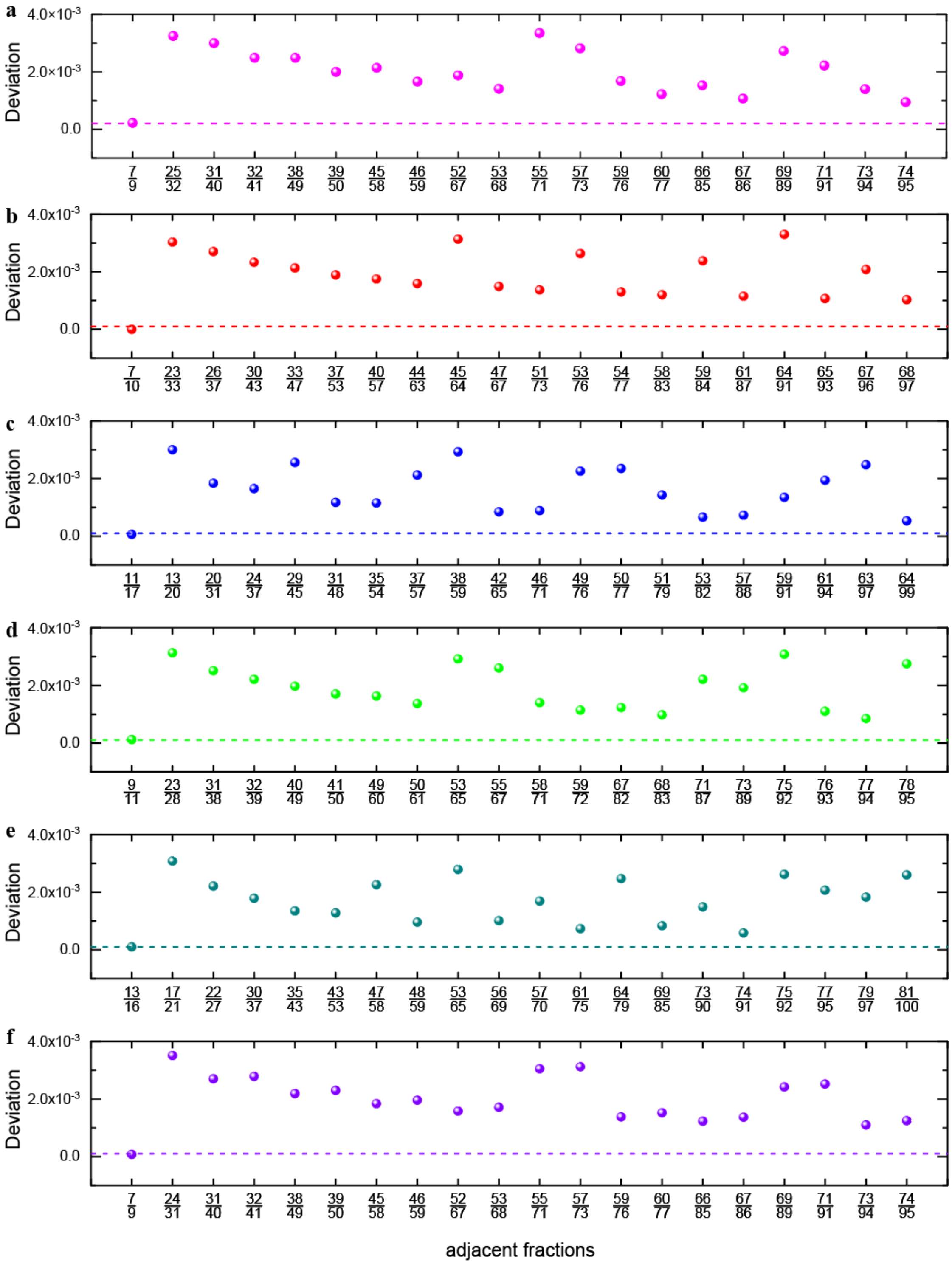

Supplementary Figure 4| Comparison between decimal values for anomalous plateaus and their adjacent fractions. The measured decimals from up to down are 0.7780, 0.7000, 0.6470, 0.8183, 0.8126, and 0.7777 in the unit of R_{K}, corresponding to plateaus in Fig. 2 with identical colors. The dashed lines are standard deviations of each plateau labeled in Fig. 2.

References

1. Buttiker, M. Four-terminal phase-coherent conductance. *Phys. Rev. Lett.* **57,** 1761-1764 (1986).
2. Buttiker, M. Absence of backscattering in the quantum Hall effect in multiprobe conductors. *Phys. Rev. B* **38,** 9375-9389 (1988).
3. Landauer, R. Conductance from Transmission - Common-Sense Points. *Physica Scripta* **T42,** 110-114 (1992).
